# Diagnostic yield of 1000 trio analyses with exome and genome sequencing in a clinical setting

**DOI:** 10.3389/fgene.2025.1580879

**Published:** 2025-06-20

**Authors:** Helena Malmgren, Malin Kvarnung, Peter Gustafsson, Britt-Marie Anderlid, Cecilia Arthur, Jonas Carlsten, Karl De Geer, Emma Ehn, Giedre Grigelioniené, Anna Hammarsjö, Hafdis T. Helgadottir, Maritta Hellström-Pigg, Erik Iwarsson, Ekaterina Kuchinskaya, Hillevi Lindelöf, Maria Mannila, Daniel Nilsson, Maria Pettersson, Eva Rudd, Ellika Sahlin, Bianca Tesi, Emma Tham, Håkan Thonberg, Eini Westenius, Johanna Winberg, Max Winerdal, Magnus Nordenskjöld, Maria Johansson-Soller, Valtteri Wirta, Ann Nordgren, Anna Lindstrand, Kristina Lagerstedt-Robinson

**Affiliations:** ^1^ Department of Clinical Genetics and Genomics, Karolinska University Hospital, Stockholm, Sweden; ^2^ Department of Molecular Medicine and Surgery, Karolinska Institutet, Stockholm, Sweden; ^3^ Science for Life Laboratory, Department of Molecular Medicine and Surgery, Karolinska Institutet, Stockholm, Sweden; ^4^ Genomic Medicine Centre Karolinska, Karolinska University Hospital, Stockholm, Sweden

**Keywords:** trio analysis, exome, genome, syndrome, *de novo*, NDD

## Abstract

**Introduction:**

A trio analysis refers to the strategy of exome or genome sequencing of DNA from a patient, as well as parents, in order to identify the genetic cause of a disorder or syndrome.

**Methods:**

During the last 10 years, we have successfully applied exome or genome sequencing and performed trio analysis for 1,000 patients.

**Results:**

Overall, 39% of the patients were diagnosed, with the detection of causative variant(s). The variants were located in 308 different genes. Autosomal dominant *de novo* variants were detected in 46% of the solved cases. Detection rates were highest in patients with a syndromic neurodevelopmental disorder (46%) and in patients with known consanguinity (59%). Even for patients previously analyzed as singletons, using a pre-defined gene panel, a consecutive trio analysis resulted in the detection of a causative variant in 30%.

**Discussion:**

A major advantage of trio analysis is the immediate identification of *de novo* variants as well as confirmation of compound heterozygosity. Additionally, inherited variants from a healthy parent can be dismissed as non-disease causing. The trio strategy enables analysis of a high number of genes–or even the whole genome–simultaneously. The strengths of a trio analysis, in combination with analysis of genome sequence data, allows for the detection of a wide range of genetic aberrations. This enables a high diagnostic yield, even in previously analyzed patients. Our current protocol for trio analysis is based on genome sequencing data, which allows for simultaneous detection of single nucleotide variants, insertion/deletions, structural variants, expanded short tandem repeats, as well as a copy number analysis corresponding to an array-CGH, and analysis regarding *SMN1* gene copies.

## 1 Introduction

Patients with congenital syndromes or rare diseases constitute a highly heterogeneous group displaying a range of symptoms. Common symptoms include global developmental delay, intellectual disability, autism spectrum disorders, and congenital malformations. In addition, many patients display seizures, brain malformations, neuromuscular disorders, and distinctive craniofacial abnormalities or dysmorphic features.

To identify the genetic cause of different symptoms in patients, exome sequencing (ES) and genome sequencing (GS) have been widely used in clinical settings, by our clinic and worldwide. In many settings, only genes known to be relevant for the symptoms are analyzed in sequenced data (ES or GS) from the patient (a gene panel). A trio analysis refers to a strategy where samples from both the patient and the healthy parents are sequenced and analyzed together, with the advantage that all genes known to cause Mendelian diseases can be analyzed simultaneously. In a clinical analysis, we focus on genes included in the OMIM morbid gene panel (genes associated with disease), while a research analysis may include all known genes in the genome. The strength of such a setup is that more genetic aberrations can be identified and classified as causative, based on their inheritance patterns, with the identification of both inherited and *de novo* variants. In addition, variants in genes associated with dominant disorders with high penetrance can be excluded and marked as non-causative if present in a healthy parent. Thus, a trio analysis enhances the probability to detect a disease-causing variant in these patients, as opposed to gene panels which targets a subset of genes linked to specific phenotypes. Disadvantages include the need to perform genetic testing on healthy individuals, with the risk for secondary/incidental findings, and a higher cost due to additional sequencing. In addition, autosomal dominant inherited causative variants can be missed due to reduced penetrance, variable expression or poorly phenotyped/clinically examined parents (Smedley et al., 2021; Sanchis-Juan et al., 2023; Wright et al., 2023).

Yang et al. (2014) reported that trio ES identified causative variants in approximately 31% of cases, underscoring its effectiveness compared to traditional methods like single-gene testing (Yang et al., 2014). Additional studies performed in children with developmental disorders have revealed a diagnostic yield spanning 27%–42% (Wright et al., 2015; DDDS, 2015), a span confirmed in more recent studies (Wright et al., 2023).

The trio approach has also been confirmed successful in identifying novel disease genes. For instance, the above-mentioned study contributed to the identification of more than 30 new genes linked to developmental disorders, significantly expanding the genetic landscape of these conditions (Wright et al., 2015; Wright et al., 2023). Furthermore, the trio analysis broadened the phenotypic spectrum associated with known disease genes. For example, variants in the *CHD7* gene, primarily associated with CHARGE syndrome (OMIM #214800), have recently been found in patients with overlapping but distinct clinical features, widening the spectrum of *CHD7*-related disorders (van Ravenswaaij-Arts et al., 2022).

At Karolinska University Hospital, trio analysis has been performed in clinical setting since 2012, initially using ES. From 2015 GS has gradually replaced ES. To date, trio analysis has been performed in a total number of 1,000 unique patients manifesting a large range of different combinations of symptoms.

The aim of this study was to determine the detection rate of causative sequence variants: pathogenic (class 5) and likely pathogenic sequence variants (class 4) that could explain the phenotypes of the patients. In addition, we also report the detection rate of variants of unknown significance (VUS, class 3), with strong clinical concordance that most possibly could explain the phenotype and that was reported to the referring physician/patient.

Another aim was to investigate specific parameters that could influence the detection rate of causative variants in patients. For example, certain symptoms or combinations of symptoms that correlate with a higher rate of identified causative sequence variants and patients/syndromes that correlate with a very low chance of identifying the genetic cause of a disorder.

In addition, an aim was to clarify if performing a trio analysis is relevant if a targeted gene panel had previously been performed.

## 2 Material and methods

### 2.1 Study subjects

The Swedish Ethical Review Authority has approved this study (Dnr 2014/983-31/1, 2012/2106-31/4), which follow the tenets of the Declaration of Helsinki. Patients and their families received oral and written study information and informed consent was obtained from the patients (if adult), or the parents (if a minor). Information included the possibility of identification of secondary findings.

All 1,000 consecutive patients included in this study were referred to the Department of Clinical genetics and genomics, Karolinska University Hospital between May 2012 - January 2023 for clinical evaluation and genetic analysis. The patients exhibited a variety of symptoms and combinations of symptoms. All trio analyses performed during the study time period were included in this study. The study includes 1,000 unique cases, some of them which have been reanalyzed during this period. The result from the most recent analysis is included in the results.

Genomic DNA was extracted mainly from peripheral blood leukocytes using standard protocols. In a few cases, DNA was extracted from fetal tissue from aborted fetuses, other tissue samples and Guthrie cards.

### 2.2 Phenotypic categorization of patients

**FIGURE 1:**
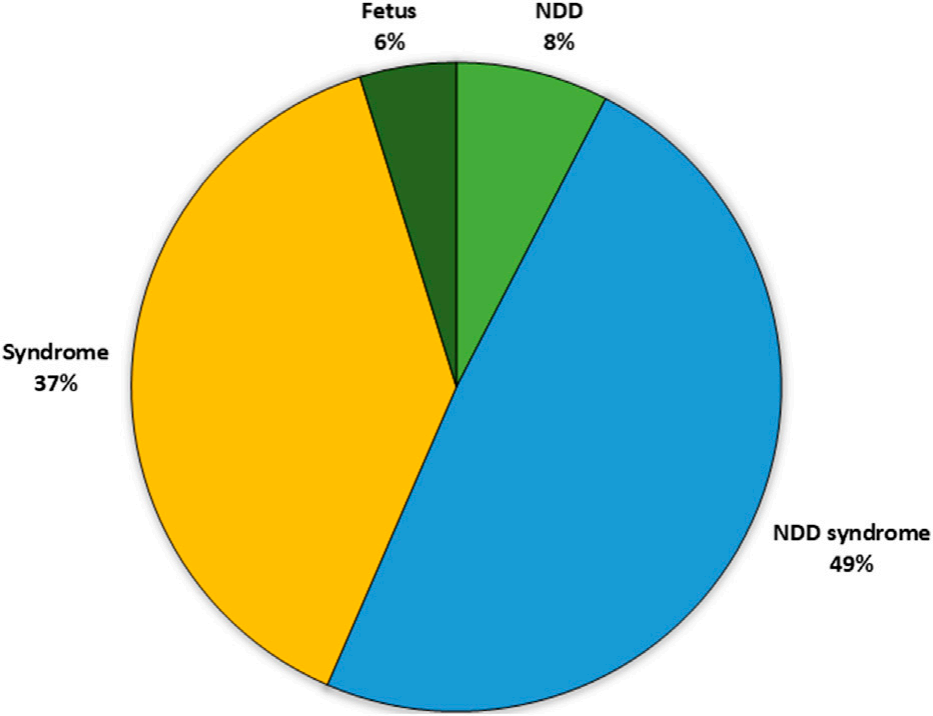
Distribution of the patient subgroups in the analyzed cohort based upon clinical phenotype. The largest cohort was the patients with syndrome including NDD (49%) followed by patients with a syndrome without NDD (37%) and patients with only NDD (8%). Finally, a small proportion of the patients were fetuses. NDD, neuro developmental disorder.

Information of the symptoms and phenotype of each patient was collected from the referral. The patients in the cohort were categorized into four different groups (Figure 1): 1) NDD (only); patients with reported neurodevelopmental disorder (DSM5), with no indication of a syndromic phenotype/other symptoms. 2) NDD + Syndrome; patients with neurodevelopmental disorder with additional symptoms indicating a syndrome 3) Syndrome without NDD; patients with no neurodevelopmental symptoms reported, but one or several other symptoms. 4) Analyzed samples retrieved from fetuses.

The categorization was mainly based on whether the patient had symptoms of Neuro-developmental Disorder (NDD, DSM5) (American psychiartic association, 2022), including any of the HPO terms (https://hpo.jax.org/app/): Neurodevelopmental delay HP:0012758, Global developmental delay HP:0001263, Motor delay HP:0001270, Delayed speech and language development HP:0000750, Intellectual disability HP:0001249, Autistic behavior HP:0000729, Autism HP:0000717.

In the cohort, other recurrent symptoms were identified using HPO terms, which described and categorized symptoms among the patients (Table 1). Essentially the explanation of each HPO term used was followed for each term (https://hpo.jax.org/app/). The HPO term “Growth abnormality” (HP:0001507) included both pre- and postnatal growth retardation, abnormality of body height and body weight and asymmetrical growth.

**TABLE 1:** Distribution of phenotypes in the analyzed cohort. n, number; NDD, neuro developmental disorder.

Patient subgroups	Categories of symptoms	Total patients (n = 1,000)	NDD (only) (n = 76)	NDD + syndrome (n = 489	Syndrome (no NDD) (n = 370)	Fetus (n = 65)
NDD (DSM5)	Neurodevelopmental delay HP:0012758 Global developmental delay HP:0001263 Motor delay HP:0001270 Delayed speech and language development HP:0000750	56.6% (565/1,000)	100% (76/76)	100% (489/489)	-	-
	Intellectual disability HP:0001249					
	Autistic behavior HP:0000729 Autism HP:0000717					
Symptoms of syndromes	Seizures HP:0001250	14.8% (148/1,000)	-	23.7% (116/489)	8.6% (32/370)	-
	Abnormality of brain morphology HP:0012443	21.3% (213/1,000)	-	26.2% (128/489)	15.4% (57/370)	43.1% (28/65)
	Microcephaly HP:0000252	10.2% (102/1,000)	-	14.3% (70/489)	6.8% (25/370)	10.8% (7/65)
	Macrocephaly HP:0000256	3.9% (39/1,000)	-	5.3% (26/489)	3.5% (13/370)	-
	Growth abnormality HP:0001507	18.9% (189/1,000)	-	22.5% (110/489)	20.3% (75/370)	6.2% (4/65)
	Abnormality of the face HP:0000271	30% (300/1,000)	-	38.7% (189/489)	25.7% (95/370)	33.8% (22/65)
	Abnormality of skeletal system HP:0000924	13.8% (138/1,000)	-	8.2% (40/489)	21.6% (80/370)	27.7% (18/65)
	Muscular hypotonia HP:0001252	12.0% (120/1,000)	-	16.6% (81/489)	10.3% (38/370)	1.5% (1/65)
	Congenital contracture HP:0002803	4.1% (41/1,000)	-	1.6% (8/489)	7.8% (29/370)	6% (4/65)
	Visual impairment HP:0000505	5.3% (53/1,000)	-	6.5% (32/489)	5.7% (21/370)	-
	Hearing abnormality HP:0000364	14.4% 144/1,000	-	9.2% (45/489)	7.3% (27/370)	-
	Abnormality of cardiovascular system morphology HP:0030680	13.3% 133/1,000	-	11.5% (56/489)	17.6% (65/370)	18.5% (12/65)
	Abnormality of kidney HP:0000077	5.1% (51/1,000)	-	3.5% (17/489)	5.4% (20/370)	21.5% (14/65)
	Congenital malformations (CM)	24.4% (244/1,000)	-	20.0% (92/489)	31.4% (116/370)	55.4% (36/65)
	Neuromuscular disease/Spastic paraparesis/Ataxia	7.1% (71/1,000)	-	8.6% (42/489)	7.8% (29/370)	-
	Other	24% (240/1,000)	-	20.7% (101/489)	35.1% (130/370)	15.4% (10/65)

In addition to the identified recurrent HPO terms, congenital malformations were present in a large number of patients. Recurrent congenital malformations, e.g., retentio testis, cleft palate, club feet, hypospadias, and coloboma. Neuromuscular disorders, spastic paraparesis, and ataxia were other recurrent reasons for referral. The list of phenotypes based on HPO terms, was thus expanded to include three additional categories: 1) Congenital malformations 2) Neuromuscular disorders/ataxia/spastic paraparesis and 3) Other (including a mix of sporadic phenotypes e g. immunological phenotypes, leukemia, primary pulmonary hypertension). The identified recurrent phenotypes and subgroups are listed in Table 1. The patients with a suspected syndrome had in most cases 1–4 of the categorized symptoms in addition to NDD. Symptoms used in this compilation were exclusively derived from referral data and were subsequently utilized in the analyzes for interpretation of sequence alterations.

It was also noted if consanguinity between the parents was specified in the referral.

### 2.3 Massive parallel sequencing and variant calling

**FIGURE 2:**
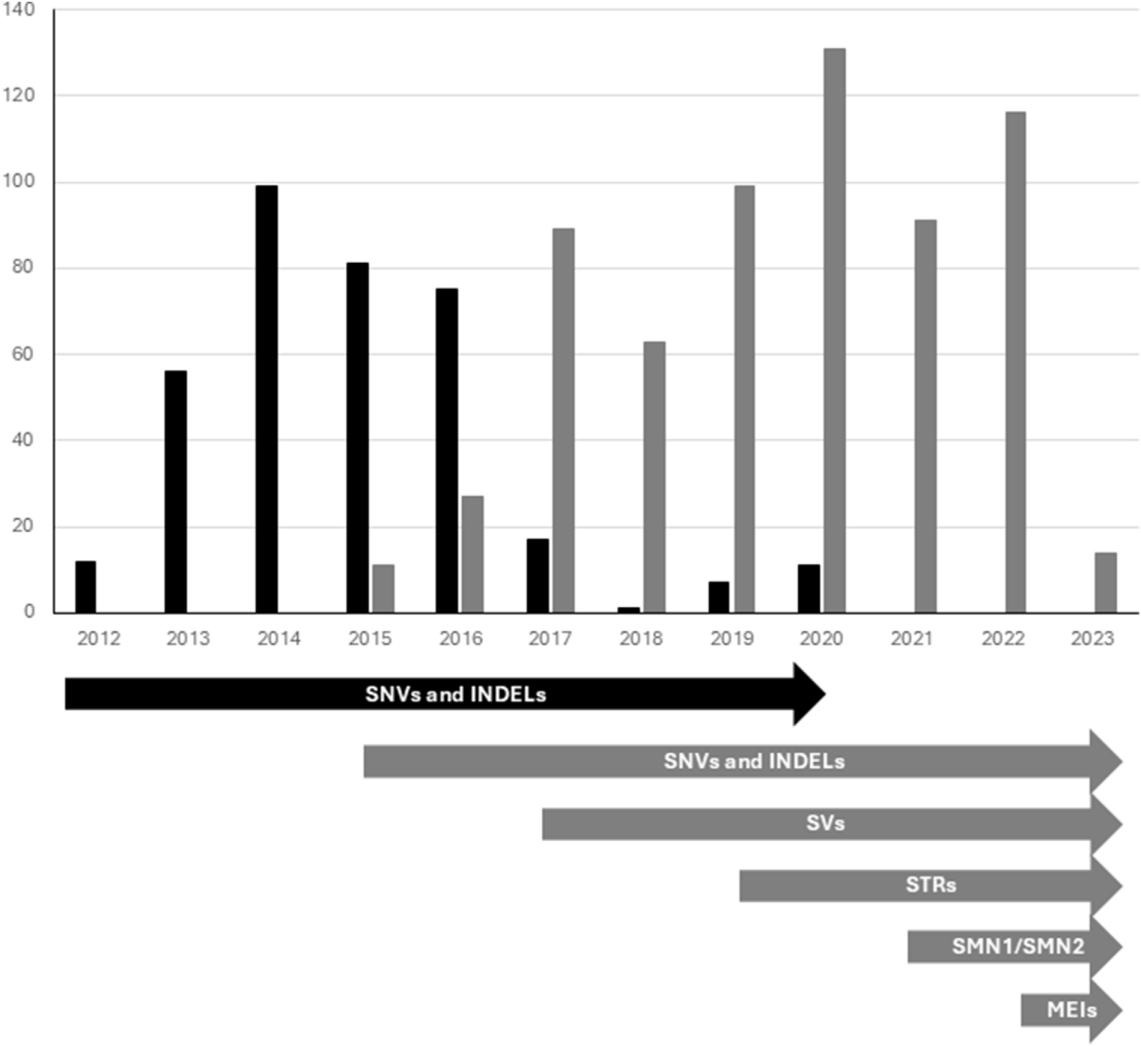
Trio based diagnostics performed by ES (black bars) and GS (grey bars) from May 2012-January 2023. Timeline showing integration of new components to the workflow (illustrated by arrows). ES, Exome sequencing; GS, Genome Sequencing; SNVs and INDELs, Single nucleotide variants and smaller insertions and/or deletions; SVs, Structural variants; STRs, Short tandem repeats; SMN, Copy number identification of *SMN1* and *SMN2* genes; MEIs, Mobile element insertions.

ES analyses performed 2012–2016 were performed at Oxford Genome Technologies (http://www.ogt.co.uk/). Briefly, paired end sequencing libraries were prepared according to the manufacturer’s instruction (TruSeq, Illumina, San Diego, CA, United states), exonic sequences captured using Agilent SureSelect AllExon v4 (Agilent) and sequenced on an Illumina HiSeq 2500 instrument in high output mode (2 × 100 bp). Reads were mapped to the hg19 reference genome using Burrows–Wheeler Aligner, and variants were called using the Genome Analysis Toolkit. During 2016 and onward ES was also performed at Clinical Genomics, Stockholm, Sweden using Agilent SureSelect All Exon v4-7, Focused Exome or Twist Human Core Exome using Illumina HiSeq 2500 or Illumina Nova Seq 6000 platform (Figure 2). Different categories of genetic variants were called using Mutation Identification Pipeline (MIP) (https://github.com/Clinical-Genomics/MIP) (Stranneheim et al., 2021).

GS was performed at Clinical Genomics, Stockholm, Sweden using the Illumina HiSeq X Ten, or NovaSeq 6000 platforms, using a 30× PCR-free paired-end WGS protocol. Genomic variants were called using MIP (Figure 2).

Variants (SNVs and INDELs) were filtered for *de novo* variants, X-linked inheritance, and autosomal recessive inheritance (homozygous or compound heterozygous) in each family. Only sequence variants with an allele frequency below 1% in the general population, affecting exons or exon-intron boundaries were taken into consideration. In the case of autosomal recessive inheritance two variants in trans in the same gene had to be present, in order to be considered a disease-causing gene.

Over the years, genome data analysis pipelines have improved (Stranneheim et al., 2021). Evolving from focusing solely on the analysis of the SNVs and INDELs, our pipeline today also includes analysis regarding structural variants (SVs) - such as deletions, duplications, and inversions (introduced 2017), analysis of repeat expansions (STRs) (introduced 2019) and incorporated specialized tests for assessing copy number variations of the *SMN1*-gene (introduced 2021) (Figure 2).

For analyses performed after 2019, GS data was also analyzed regarding structural variants (SVs), short tandem repeats (STRs) and *SMN1* copy number determination. If array-CGH had not been performed previously, vcf-files were imported into CytoSure software (Oxford Gene Technology), for an analysis corresponding to an array-CGH, with the resolution of about 30 kb (Lindstrand et al., 2019). Most of the patients included in this study had previously been analyzed with array-CGH or a GS analysis corresponding to an arrayCGH (performed when analyzed as a singleton with a gene panel).

The genes included in the analysis were all OMIM Morbid genes known at the time of analysis. When this study started the number of genes analyzed was around 2,700 (Hamosh et al., 2021) and today this number is 4,800 (https://omim.org/statistics/geneMap).We also have the possibility to reanalyze ES or GS data and include all genes, if consent from the family has been given.

The genetic results were assessed by a clinical laboratory geneticist together with a specialist in Clinical Genetics, usually the physician that had met the patient, to identify the genetic alteration causing the symptoms in the patient.

Most of the reported variants were verified using an alternative molecular method, Sanger sequencing for SNVs and INDELs, MLPA or array-CGH for larger deletion and duplications (SVs) or fragment length analysis for STRs. Regarding Sanger sequencing, the regions of interest were amplified using specific primers. Direct Sanger sequencing was performed on both strands using Big-Dye terminator sequencing (v1.1, Applied Biosystems, CA, United states), and run on an ABI3500XL Genetic Analyzer (Applied Biosystems, CA, United states). The sequencing reactions were carried out according to the manufacturer’s recommendations. Chromatograms were analyzed using SeqScape v3.7 (Applied Biosystems, CA, United states) using the NCBI RefSeq library as a reference sequence (hg19).

The variants that were considered likely causative for the symptoms of the patient, and that were reported to the referring physician/patient, were classified according to ACMG guidelines (Richards et al., 2015). These variants were ACMG class 4 or 5, in a gene with a corresponding phenotype that was in consistence with the symptoms of the specific patient. In some cases, a variant of unknown significance (VUS), ACMG class 3, was reported to the referring physician/patient, as the phenotypic correlation between the patient and the described syndrome was strong. In these cases, segregation of the variant in the family or additional clinical examination of the patient was recommended.

## 3 Results

### 3.1 Diagnostic rate

In total, 1,000 unique trio analyses were performed, yielding likely causative sequence variants in 393 cases (39%). Of the 1,000 trio analyses, 359 (36%) were performed using ES, including Agilent Focused Exome (n = 50) and Twist Human Core Exome (n = 2). GS were performed in the remaining 641 cases (Figure 2). The hit-rates of ES versus GS differed slightly (36% versus 40%).

**FIGURE 3:**
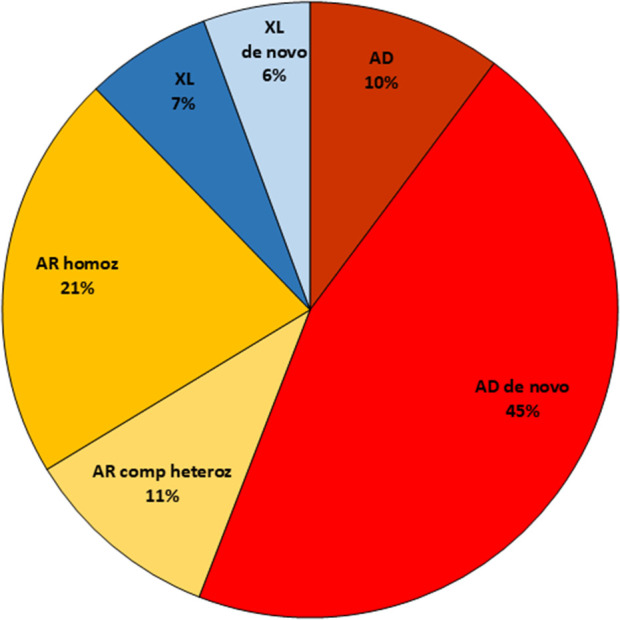
Illustration and distribution of the different inheritance patterns among solved cases in the study. AD, Autosomal dominant; AR, Autosomal recessive; XL, X-linked.

The investigated patients were predominantly males 59% (548/935) and 41% females (387/935). The degree of solved cases was about the same for both sexes (41% for males and 38% for females). The distribution of inheritance patterns in the solved cases was equal for both sexes, except for the X-linked disorders. The X-linked causative variants identified in the female cohort were predominantly *de novo* (14/16 cases), while in males X-linked recessive variants inherited from a mother were detected in 78% (25/32), see Figure 3. The cohort also included 65 samples from affected fetuses (terminated or ongoing pregnancies), where abnormalities had been detected by ultrasound investigations. The diagnostic yield was slightly lower in this group (18/65 cases; 27%).

### 3.2 Inheritance

Among the 393 solved cases, autosomal dominant *de novo* variants were detected in 177 cases (45%), and X-linked *de novo* in 23 cases (6%), giving a total of 51% *de novo* events. Autosomal recessive inheritance was observed in 125 cases (32%) with 83 homozygous cases. X-linked inheritance, where the mother was a heterozygous carrier, was detected in 27 cases (7%). In two of these 27 cases, the patient was a girl. In one of these two cases, skewed X-inactivation was detected in the healthy mother. In the other case, no skewed X-inactivation could be detected in the mother or in the patient. A few variants with autosomal dominant inheritance were reported (n = 40, 10%), with either a known affected parent, reduced penetrance in the parent or where parental mosaicism or germinal mosaicism was detected (Figure 3).

Consanguinity was noted in the referrals for 103 cases, of which 61 (61/103; 59%) genetic causative variants were identified; 55 cases with homozygous variants (including one case that also had a *de novo* variant) and five cases of *de novo* variants, of which four cases were autosomal dominant and one case displayed X-linked inheritance. Of all disease-causing homozygous variants (n = 83) in the entire cohort, 65% were detected in the known consanguineous families.

Nineteen of all cases received a possible dual diagnosis, i e variants in two different genes were reported to the referring physician/patient. This implies either two diagnoses in these patients or that genotype/phenotype could not be distinguished between the different entities, thus requiring further analyses. In nine of these cases, two homozygous variants in two different genes were implied. In total two-thirds of these probands were from consanguineous families. Three of the cases had a *de novo* variant, but also two variants (homozygous or compound heterozygous) corresponding to an autosomal recessive inheritance. Three cases were males where the two reported variants were X-linked and inherited from the mother. The remaining four cases constituted two cases with two *de novo* variants and two cases of an autosomal recessive disorder combined with an autosomal dominant disorder trait inherited from a parent. The exact number of cases with symptoms corresponding to a dual diagnosis is unknown, as we do not have access to follow-up and renewed clinical information in all cases.

We also identified two cases of germinal mosaicism where the variant was identified in a sibling or at prenatal diagnosis of an ongoing pregnancy, respectively. The variant could not be detected in DNA isolated from blood from either parent.

### 3.3 Reanalysis of data

**FIGURE 4:**
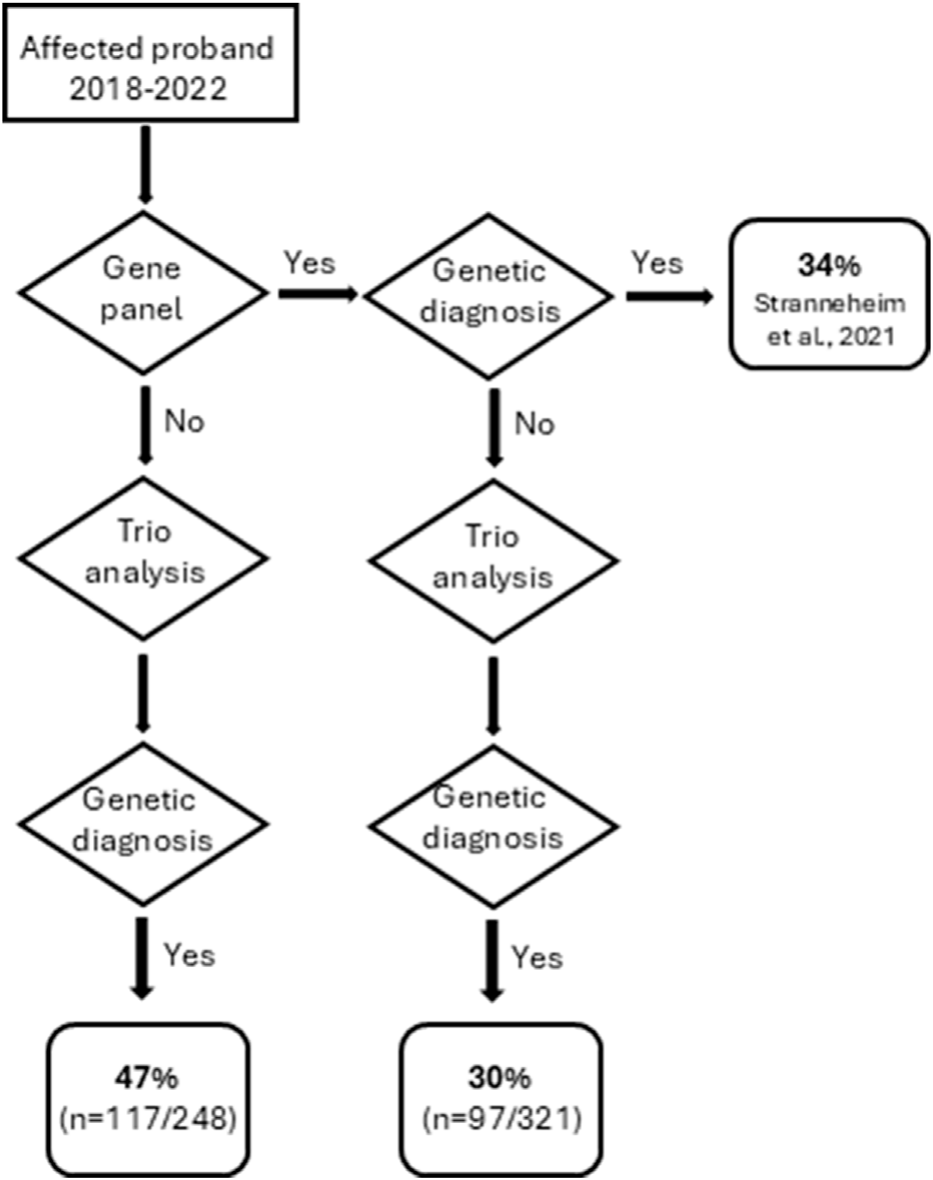
Flowchart describing the outcome of trio analyses performed between January 2018- January 2023.

Reviewing the 569 patients referred for trio-analysis in the five most recent years (January 2018 – January 2023), the diagnostic yield was in total 38% (214/569). In this cohort, 44% (248/569) had no previous ES or GS analysis performed, and the diagnostic yield of theses trio analyses was 47%. For 56% (321/569), a previous analysis using specific gene panel(s) (based on patient phenotype), or a previous trio analysis had been performed, without detecting a causative variant. The trio analysis of this subgroup of 321 patients still resulted in the detection of a causative variant in 30% of the cases (Figure 4).

Reanalysis of existing trio data was performed in 97 cases in total (approximately 10% of all 1,000 cases) during the study timeframe (May 2012 – January 2023). A causative variant was reported in 33% of these cases (32/97). The results from the reanalysis are included in the overall summary of this study.

### 3.4 Genes, variants and classification

The number of solved cases was 393, and in total 443 reported causative variants were classified according to ACMG criteria at the time of reporting. The different reported variants were of class 5 (24%), class 4 (46%), or class 3 (30%). Class 3 variants were reported only if assessed as likely pathogenic from a clinical perspective (so called class 3+). These cases were discussed in multidisciplinary team meetings; the criterium used for categorizing a gene variant as class 3 + was a relatedness between the phenotype of the patient and the description of the disorder based on known literature. The inheritance patterns for the 136 class 3+ variants were autosomal recessive homozygous (33%), compound heterozygous (30%), autosomal dominant (20%) or X-linked (17%).

In total, causative sequence variants in 308 different genes were identified. The most common gene was *MECP2* identified in eight cases with X-linked *MECP2*-related disease/Rett syndrome (OMIM *300005/#312750) followed by *ANKRD11* identified in seven cases consistent with autosomal dominant KBG syndrome (OMIM #148050). *ARID1B* and *HUWE1* were each reported in five cases, followed by *DMPK, DYNC2H1, GRIN2B, MYH3* and *NSD1* each detected in four cases. The vast majority of all genes (254/308, 82%) were reported only in one single case.

Certain variants were recurrently detected in different cases; *ALDH5A1* (c.1402 + 1G>T, AR homozygous inheritance), *ALG9* (c.660 + 2T>A, AR homozygous inheritance), *BMP2* (gene deletion, AD inheritance, and one case *de novo*), *BRAT1* (c.294dup, p.(Leu99Thrfs*92) AR inheritance where the compound variant varied between the two cases), *CLCN4* (gene deletion, X-linked inheritance), *KDM5C* (c.1757C>A, p.(Thr586Lys) X-linked *de novo* inheritance) and *TTI2* (c.1048A>G, p.(Met350Val) AR homozygous inheritance).

Missense variants were the most common variants, in total 231 different missense variants were reported. The number of loss-of-function variants added up to 126 (n = 60 frameshift variants, n = 66 nonsense variants). Variants possibly affecting splicing were reported 42 times. The remaining variants were deletions/duplications of one or more amino acids, variants affecting the first or the last codon, larger deletions or duplications as well as expansions (i.e., in the *DMPK* gene).

Excluded from this summary are variants in genes presently not described with a known phenotype, but where the inheritance pattern in the trio, and/or function of the gene, makes it a very strong candidate for observed symptoms. In total we have 11 such genes/variants.

### 3.5 Phenotypes and results

The majority of patients referred for trio analysis had neurodevelopmental disorders (NDD) (565/1,000; 56%) and in most of these cases (86%), additional clinical features were present. The frequency of identified causative variant(s) varied between the phenotypic subgroups (Table 2). In total, 44% of all cases in the NDD group were solved. Among patients with isolated NDD (8% of the cohort), the rate of solved cases was 33%. In the subgroup NDD + Syndrome (49% of the cohort), 46% of the cases were solved. In the patient group without NDD, Syndrome (37% of the cohort), causative variants were detected in 34% of the cases. Of all the solved cases (251/393), 64% had NDD.

**TABLE 2:** Subgroups of the cohort of 1,000 patients and results. n, number; NDD, neuro developmental disorder.

Cohort (n = 1,000)	n	% Of cases	Solved cases (n = 393)	% Solved cases
NDD (all)	565	56%	251	44.4%
-NDD (only)	76	8%	25	32.8%
-NDD + Syndrome	489	49%	226	46.2%
Syndrome without NDD	370	37%	124	33.5%
Foetuses	65	6.5%	18	27.7%

Additional symptoms of the patients included in this cohort were registered, and recurrent symptoms/HPO terms identified. Some patients only displayed one of the phenotypes, but others have multiple symptoms with a complex phenotype. About 30% of the patients in the subgroups NDD + Syndrome, Syndrome, and Fetus were described having one symptom, ∼30% having two, ∼25% having three, and the remaining 15% having >3 symptoms (NDD excluded). In the subgroup NDD + Syndrome, the most common additional symptoms were seizures, abnormality of brain morphology, growth abnormalities, and dysmorphic features. In patients with a syndrome without NDD, the most common symptoms were congenital malformations, dysmorphic feature, skeletal abnormalities, and other. In the fetuses the major phenotypes were congenital malformations, abnormal brain morphology, dysmorphic feature, and skeletal abnormalities.

## 4 Discussion

### 4.1 Diagnostic rate

In this study we have collected 1,000 unique trio analyses performed over a ten-year time span. In total, a likely causative variant explaining the patient’s phenotype was reported in 39% (n = 393) of the cases. This is a result comparable to the large study of 13,610 cases performed by Genomics England (Wright et al., 2023) where 41% of the probands received a clinical diagnosis. That study was based on clinically specific gene panels, and not the whole OMIM morbid gene panel as we have analyzed in this study.

In a previous study from our clinic, patients with NDD analyzed with only array-CGH and the *FMR1* gene, were shown to have a diagnostic yield of 11%. Adding a gene panel for intellectual disability, the diagnostic yield increased by 26% (Lindstrand et al., 2022).

In this study, the trio analysis of the patient was often performed as a follow-up after a specific gene panel had been performed and analyzed (320/568 trios: 56%) during the years 2018–2022. It follows that patients analyzed as singletons, and solved using specific gene panels are not included in the present study. Our diagnostic rate for singletons with heterogeneous symptoms, analyzed with a specific gene panel, has previously been reported to be 34% (Stranneheim et al., 2021).

If looking at trio analyses performed within the last 5 years (2018–2022), the diagnostic rate was 47% if the patient had not been previously analyzed. It should be noted that for cases where a previous gene panel - or in rare cases a previous trio analysis - had been performed before the most recent trio analysis, the diagnostic yield for these patients was still as high as 30% (Figure 4). This went against our expectations to rather see a significantly lower diagnostic yield from the trio analyses in cases where the proband previously had been subjected to a clinically specific gene panel(s) before the analysis.

### 4.2 Genes, variants and classification

At the onset of the studied period, in 2012, the analysis was performed using ES, which over time was increasingly replaced by GS. One advantage of GS is the possibility to detect different kinds of sequence variants. For instance, analysis regarding structural variants was introduced in 2017, causing the large shift this year. Similarly, analysis regarding expansion of repetitive elements, for example, the one causing myotonic dystrophy type 1 (OMIM #160900, *DMPK*-gene) was introduced in 2019 (Stranneheim et al., 2021) (Figure 2). In total, 359 cases (36%) were performed using ES and the remaining 641 (64%) cases performed using GS. The hit-rate differed slightly between ES and GS (36% versus 40%). This difference in hit-rate is partly explained by the progress of analysis tools for GS data. Arguably, the difference in hit rate could be expected to be even higher, as structural variants (SV) are expected to be identified in ∼10% of patients with different syndromes. In general, array-CGH, or a comparable GS-analysis (since 2019) has been performed on the patient sample before trio analysis, and patients already solved by these analyses are not included in this study. This may explain why we only have identified a handful of structural variants in the present study.

It should be taken into consideration that the possibility of analysis of GS of larger deletions or duplications, as well as expansions, was introduced and put into wider practice in 2017 and 2019 respectively. In other words, not all cases were analyzed using the same repertoire of genetic bioinformatic tools. Since 2019, a trio analysis performed at our clinic includes interpretation of SNV/indels, structural variants, repeat expansions as well as using GS data for analysis corresponding to an array-CGH (if not previously performed).

Another explanation to higher detection rate when performing GS is the increased knowledge of disease associated genes over time. Cases performed with ES were conducted many years ago, with fewer known disease-causing genes at that time. Reanalysis of data might be relevant simply because more known disease-causing genes can be evaluated today.

All reported variants were classified according to ACMG (Richards et al., 2015) at the time of reporting. Although our main goal is to report variants that are pathogenic (class 5) or likely pathogenic (class 4), 30% of our variants were classified as unknow clinical significance (class 3). Class 3 variants were reported only if assessed as likely pathogenic from a clinical perspective (so called class 3+). The inheritance patterns for these variants were mainly autosomal recessive (63%) followed by autosomal dominant (20%) and X-linked (17%) inheritance. In general, it is very challenging to classify a variant as class 4 or class 5 variant if the reported variants are not *de novo* or have not been described before in patients. In addition, most genes lack an easily available functional test to further validate its pathogenicity. As we are dealing with rare disorders and rare variants it is very uncommon that the detected variants have been described in other patients at the time of reporting the findings.

### 4.3 Inheritance

Although we have not actively searched for autosomal dominant inherited variants, we have found a quite substantial number. In total 10% (40/393 solved cases) variants were reported with autosomal dominant inheritance; with either an affected parent, reduced penetrance in the parent or detection of somatic or germline mosaicism in one of the parents. In our bioinformatic scoring system of the filtered variants, a variant that previously has been reported as pathogenic (and only pathogenic) in the ClinVar database, will render a score that suggests a higher probability of the variant to be disease causing. Therefore, such a variant will be highlighted, even if inherited. In addition, four cases were detected with an expansion in the *DMPK*-gene, all inherited from the probands mothers where variable expression is known to occur depending upon the number of repeats in the *DMPK*-gene. When analyzing for expanded STRs, filtering for inheritance patterns is not performed, and thus inherited expansion will be detected. In two other cases, one of the parents were found to be mosaic for the disease-causing variant. The variants were initially detected as a *de novo* variant, but upon closer examination of the data on the parents, a few reads carrying the variant could also be detected. These cases included one maternal and one paternal mosaic case.

### 4.4 Reanalysis of data

Existing trio data were reanalyzed for 97 cases (approximately 10% of all cases) in the study timeframe. Of these reanalyzes, a causative variant was reported in 32/97 cases (33%). The results from the reanalysis are included in this study with each patient only registered as one case. The main reason why a genetic cause could be identified in the reanalysis, as opposed to the first analysis, was the inclusion of the reported gene in the updated OMIM morbid gene panel used for the analysis. Other explanations were the widening of the described phenotype during these years as well as inclusion of more inheritance patterns regarding the gene. In one case (a baby), a *de novo* variant was detected in the first analysis but not reported out since, at that time, it did not correlate with the described phenotype of the patient. Later, when the child was older, he manifested more of the features included in the syndrome, and thus the variant was then reported. None of the reported variants upon reanalysis were identified due to integration of new bioinformatic tools in our analysis pipeline, as all variants were SNVs. In summary, it is well worth to reanalyze the GS data, although we generally recommend waiting 3–5 years between the initial analysis and the reanalysis to allow the number of genes in the OMIM morbid gene panel to increase with at least hundreds of more genes compared to when the first analysis was performed. This has also been shown by others (Wright et al., 2018; Bullich et al., 2022; Laurie et al., 2025).

We have the possibility to reanalyze ES or GS data and include all genes, if consent from the family has been given. This makes it possible to discover new disease-causing genes. We have during the timeline of this study, discovered genes and variants that at the time of detection were not yet described to be associated with an established syndrome. Thus, we have identified novel disease genes or widened the phenotype for e g *ADNP* (Helsmoortel et al., 2014), *KAT6A* (Tham et al., 2015), *ALG9* (Tham et al., 2016), *USP9X* (Reijnders et al., 2016), *SATB2* (Bengani et al., 2017), *ALG14* (Kvarnung et al., 2018), *WRAP53* (Bergstrand et al., 2020) and *RBMX* (Johansson et al., 2024). These results further verifies that trio analysis is an effective way of identifying new disease-causing genes.

### 4.5 Phenotypes and results

Information of the symptoms and phenotype of each patient was only collected from the referral. We did not search medical record for confirmation or identification of additional symptoms. This might have had an impact of the subgrouping of the cohort, as we cannot be sure that all the symptoms of the patient were included in the referral. We have not used a structured phenotype protocol for the referring physician, and therefore we translated the described symptoms into HPO terms. In order not to end up with an endless number of sub-groups, we chose a high-level term in the HPO tree (Table 1). Any description of symptoms that was translated into Neurodevelopmental delay HP:0012758, Global developmental delay HP:000126, Motor delay HP:0001270, Delayed speech and language development HP:0000750, Intellectual disability HP:0001249, Autism HP:0000717, or Autistic behavior HP:0000729 resulted in the inclusion in the major group of patients with Neuro Developmental Disorder (NDD) according to DSM5.

In the present cohort, 565 (56%) patients had NDD, and in these patients we could detect a causative variant in 251 cases (44%). Of all the solved cases 251 of 393 (64%) had NDD. Most of the patients with NDD (489/565; 86%) had, in addition to NDD, other phenotypes present in different combinations indicating a syndrome (NDD + Syndrome). The patients with NDD and no additional symptoms were a minority (76/565; 14%). An explanation to this is that at our clinic, patients with isolated NDD are primarily analyzed as singletons using a pre-defined gene-panel including analysis of data corresponding to an array-CGH, with a detection rate of ∼40%. Unsolved cases are not by default extended to trio analysis, especially if the patient has only mild developmental delay, mild intellectual disability, and/or autism. However, if analyzed by trio analysis, a causative variant is identified in 33% of the cases. We detected the highest rate of solved cases (46%), in patients with both Neurodevelopmental disorder and syndrome (NDD + Syndrome).

The subgroup of fetuses had the lowest detection rate of causative variants. An explanation to this could be that the phenotype of a fetus does not completely correspond to the phenotype in a born child and many of the symptoms are not detectable in an ultrasound investigation. Thus, the genotype-phenotype correlation is more difficult to interpret.

For the consanguineous cases we identified a potential causative variant in 59%. However, most of these reported variants were class 3+ variants, but with high concordance of phenotype between the patient and diagnosis emphasizing the importance of a thorough clinical examination.

During the study period, we have reported a few incidental findings. These variants have been well known pathogenic variants predisposing to mainly either a high risk of developing cancer or a severe heart disorder where an established clinical surveillance program or a pharmaceutical treatment is available. In our bioinformatic scoring system, a variant that previously has been reported as pathogenic (and only pathogenic) will be highlighted, even if inherited. In total in our 1,000 families, we detected and reported six incidental findings. Two of these cases harbored pathogenic variants in the *BRCA1* gene thus having a high risk for hereditary breast and ovarian cancer (OMIM #604370) (Petrucelli et al., 2025). The other findings were pathogenic variants in genes causing severe heart disease (*ACVRL1, PKP2* and *SCN5A*). Among these cases was also an individual with an increased risk of malignant hyperthermia (OMIM #145600, *RYR1* gene).

A clinical trio analysis is focused on detecting variants in genes with a known genotype/phenotype correlation. The strength of the analysis is the possibility to analyze all OMIM genes in a single analysis, due to the possibility of filtering variants based on inheritance patterns. In addition, with a consent from the family, ES/GS data can be reanalyzed and include all genes in the genome. However, the clinical analysis only targets variants that we today can interpret to cause disease, i.e., variants affecting the coding part or splice regions of the genes. There are of course disease-causing variants located deep in the intron or in the UTRs of a gene, that for instance may affect the level of expression or splicing, but these variants are today hard to interpret at genomic level. It is known that adding transcriptome data to the analysis will increase the diagnostic yield (Cummnings et al., 2017; Lee et al., 2020).

Reduced penetrance and/or variable expression is a problem when analyzing trios since we assume that the parents are healthy and only analyze for Mendelian inheritance patterns with this notion as a basic requirement/postulation. In our bioinformatic setting, we have tried to address this issue by highlighting previously known pathogenic variants in ClinVar, disregarding the inheritance pattern. This will of course only add the known pathogenic variants, and not rare familial variants causing disease, as shown for instance analyzing the *ARFGEF1* gene (Thomas et al., 2021).

Trio analysis with short read sequencing will not detect epigenetic aberrations. However, epigenetic signatures have shown to be beneficial for solving NDD cases (Levy et el., 2021). We are currently in the process of introducing long-read sequencing in our clinical setting. This technique allows simultaneous methylation analysis, including detection of X-inactivation pattern, in the sequencing data and are also presumed to increase the number of detected SVs (Eisfeldt et al., 2025).

We expect that the introduction of whole transcriptome sequencing as well as methylation studies and increasing knowledge of bi-allelic inheritance/polygenic inheritance will increase the number of solved cases in these families.

When offering genetic analysis to a patient with a suspected genetic disorder or syndrome, there are different strategies which all have their pros and cons. The advantage of singleton gene panel analysis of the patient (as compared to trio analysis), is cost (one GS/ES analysis), no need for sampling of parents (they might not even be available), and reduced risk for incidental/secondary findings. However, if performing a trio analysis, all OMIM genes or even all genes in the genome can be included for analysis, due to the possibility to filter for inheritance patterns. The variants detected are easier to interpret. The number of detected variants of unknown significance (VUS) can be reduced, because variants inherited from a healthy parent is most probable rare normal variants. A trio analysis also enables the identification of *de novo* variants as well as instant confirmation of compound heterozygosity. Trio analysis also allows for the identification of genetic variants in genes with weaker links or today even no link to human disease. However, one should be aware of that variants with reduced penetrance/variable expressivity might be undetected.

## 5 Conclusion

For young patients with NDDs and/or congenital syndromes, we recommend GS and trio-analysis as a first line analysis. Compared to a singleton analysis, interpretation of data is faster and more robust thanks to the simultaneous parental status for each of the detected variants. Even more importantly, diagnostic yield of trio-analyses greatly surpasses that of singleton analyses (47% vs. 34%).

The value of trio-analysis is substantial also for patients that have had a phenotype-specific gene panel performed previously, as the detection rate of a causative variant is as high as 30% in these cases.

Lastly, reanalysis of a previous trio is relevant to conduct after a few years, as new disease-associated genes are identified each year.

Altogether, the advantages of trio-analysis lead to a more time efficient analysis with higher diagnostic yield and better quality compared to a singleton analysis.

## Data Availability

The original contributions presented in the study are publicly available. This data can be found here: Clinical Genetics and Genomics - Submitter - ClinVar https://www.ncbi.nlm.nih.gov/clinvar/submitters/505315/. The ethical approval did not permit sharing of ES or GS data, and the in-house databases used in this article are not publicly available, Requests to access the datasets should be directed to the corresponding author.

## Supplementary material

SUPPLEMENTARY TABLE SA List of disease-causing genetic variants that were identified and reported in the cohort.
